# Systematic analysis of Long non-coding RNAs reveals diagnostic biomarkers and potential therapeutic drugs for intervertebral disc degeneration

**DOI:** 10.1080/21655979.2021.1950258

**Published:** 2021-08-17

**Authors:** Jiawen Zhan, Shangquan Wang, Xu Wei, Minshan Feng, Xunlu Yin, Jie Yu, Tao Han, Guangwei Liu, Wangwen Xuan, Xiaobo Wang, Rui Xie, Kai Sun, Liguo Zhu

**Affiliations:** a General Orthopedic, Wangjing Hospital of China Academy of Chinese Medical Sciences, Beijing, China; b Scientific Research, Wangjing Hospital of China Academy of Chinese Medical Sciences, Beijing, China; c Spine Department2, Wangjing Hospital of China Academy of Chinese Medical Sciences, Beijing, China; d Orthopedic, Tianjing University of Traditional Chinese Medicine, Tianjin, China

**Keywords:** Intervertebral disc degeneration, lncRNA, support vector machine, weighted gene co-expression network analysis

## Abstract

Long non-coding RNAs (lncRNAs) are related to a variety of human diseases. However, little is known about the role of lncRNA in intervertebral disc degeneration (IDD). LncRNA expression profile of human IDD were downloaded from Gene Expression Omnibus (GEO) database. Potential biomarkers and therapeutic drugs for IDD were analyzed by weighted gene co-expression network analysis (WGCNA), R software package Limma, Gene Ontology (GO) and Kyoto Encyclopedia of Genes and Genomes (KEGG). We identified 1455 differentially expressed genes and 423 differentially expressed lncRNAs. Twenty-six co-expression modules were obtained, among them, the tan, brown, and turquoise modules were most closely related to IDD. The turquoise module contained a large number of differential expressed lncRNAs and genes, these genes were mainly enriched in the MAPK signaling pathway, TGF-beta signaling pathway. Furthermore, we obtained 11,857 LmiRM-Degenerated, these lncRNAs and genes showed higher differential expression multiples and higher expression correlation. After constructing a disease-gene interaction network, 25 disease-specific genes and 9 disease-specific lncRNAs were identified. Combined with the drug-target gene interaction network, three drugs, namely, Calcium citrate, Calcium Phosphate, and Calcium phosphate dihydrate, which may have curative effects on IDD, were determined. Finally, a genetic diagnosis model and lncRNA diagnosis model with 100% diagnostic performance in both the training data set and the validation data set were established based on these genes and lncRNA. This study provided new diagnostic features for IDD and could help design personalized treatment of IDD.

## Graphical abstract

**Figure:**
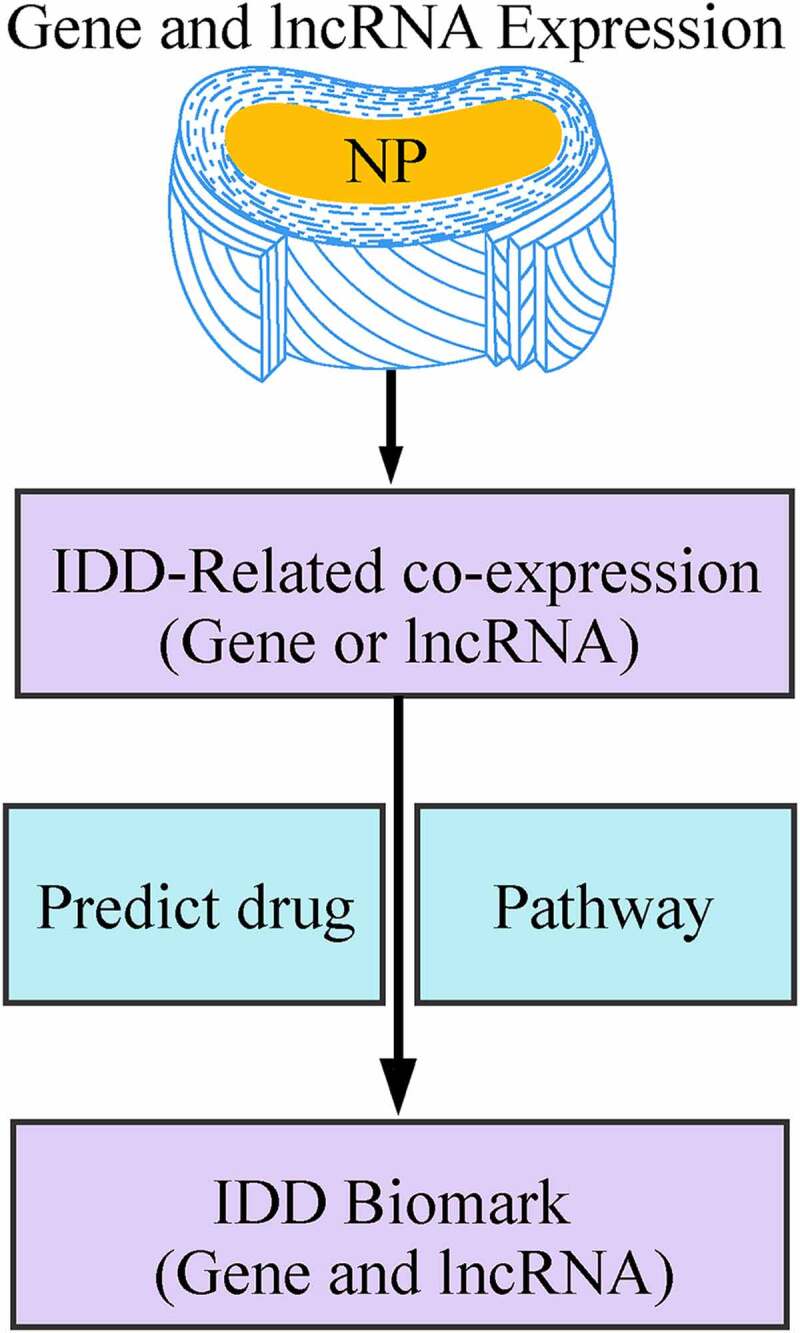

## Introduction

Low back pain (LBP) accounts for 10.7% of the total disabled population and is the most common cause of disability in developed countries [1]. In the United States, the three-month prevalence rate is as high as 40%, and 20–33% of patients are incapacitated [1]. Intervertebral disc degeneration (IDD) is a chronic disease that slowly degrades the content of intervertebral disc (IVD), which could lead to unstable IVD, thereby limiting the mobility of the spinal cord [2]. Numerous studies have shown that many cellular events take place in the IDD process from matrix synthesis to cytokine expression [3]. The basis of these changes is the dysregulation of gene expression of specific molecules. Large-scale gene expression studies have shown that many coding genes are differentially expressed in IDD, and some of them have been proven to play an important role in IDD [4,5]. The development of genetic and proteomics tools has greatly expanded our understanding of gene disorders in IDD. Several therapeutic strategies for targeted gene disorders have been presented with encouraging results in IDD animal models [6,7]. As dysregulation of gene expression is a very complicated process, previous studies have also shown that changes in several different levels of regulatory factors could ultimately result in gene dysregulation [8]. Among these factors, abnormally expressed regulatory non-coding RNAs have attracted considerable research attention in recent years.

Long non-coding RNAs (lncRNAs), defined as RNA transcripts of more than 200 base pairs in length, are a major class of ncRNAs [9]. Abnormal expression of lncRNA is closely related to human complex diseases. Dysfunction of lncRNAs contributes to the occurrence, development and metastasis of cancers [10]. For example, LncRNA UCA1 promotes the proliferation of HNSCC and cisplatin resistance through inhibiting the expression of miR-184 [11]; lncRNA EGFR-AS1 mediates epidermal growth factor receptor to regulate therapeutic response of HNSCC [12]; STAT3/HOTAIR signal regulates the growth of HNSCC in an EZH2-dependent manner [13]. Up-regulated RP11-296A18.3 may induce overexpression of FAF1, and ultimately promote abnormal apoptosis of intervertebral disc cells [14]. The expression profile showed that the expression of lncRNA in IDD is highly abnormal, indicating that lncRNA may be used as a biomarker for predicting clinical outcome.

The purpose of this study was to use the RNA expression profiles derived from IDD patients to study the potential functions of lncRNA and mRNA expression in IDD. We systematically analyzed the lncRNA and miRNA expression profiles between IDD and healthy patients. In addition, we proposed a new algorithm to identify dysregulated competitive endogenous lncRNA-miRNA-mRNA triads (LmiRM-Degenerated) during IDD progression so as to detect lncRNAs for IDD diagnosis and prognosis biomarkers and identify potentially effective therapeutic drugs.

## Methods

**Figure 1.**
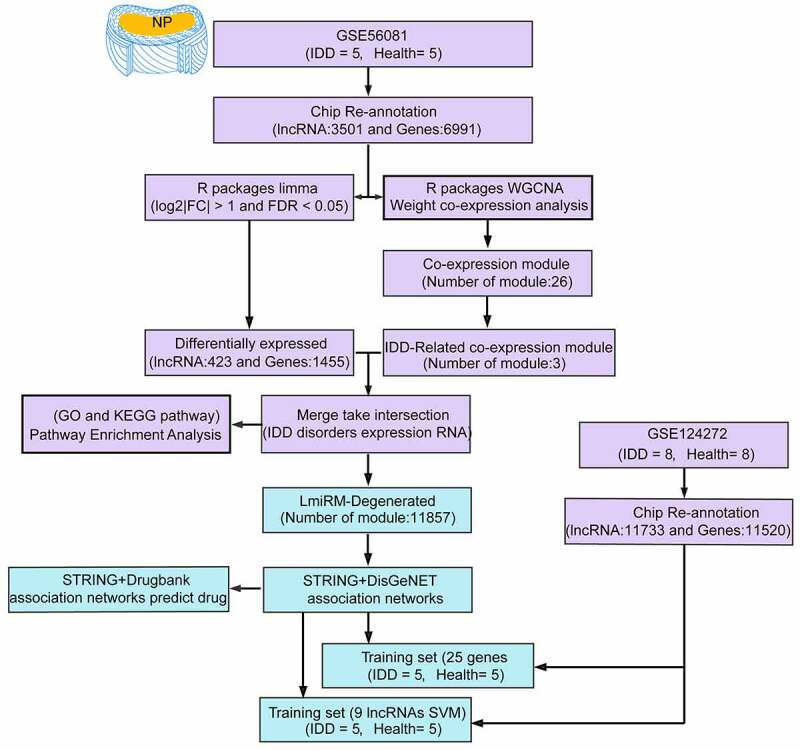
Work flow chart

The workflow is shown in Figure 1. In the workflow, the potential functions of lncRNA and mRNA in IDD were studied from the RNA expression profiles of IDD patients in GSE56081 dataset (DNA microarray datasets), and the expression profiles of lncRNA and miRNA between IDD and healthy patients were systematically analyzed.

## RNA expression profile

LncRNA expression profile of human IDD (GSE56081) was downloaded from the Gene Expression Omnibus (GEO) database (http://www.ncbi.nlm.nih.gov/geo/) [14] on the platform of Arraystar Human LncRNA microarray V2.0 (Agilent_033010 Probe Name version). The data set GSE56081 had 10 samples, including 5 IDD patients and 5 normal controls. In addition, GSE124272 [15] with 8 IDD samples and 8 control samples were used as validation dataset (the platform was Agilent-072363 SurePrint G3 Human GE v3 8x60K Microarray 039494). The GSE150408 [16] dataset from the Agilent-072363 SurePrint G3 Human GE v3 8x60K Microarray 039494 platform was also incorporated as an additional external validation set. The expression profile data of 17 IDD samples and 17 control samples were extracted. The sample information of each dataset is shown in Table 1.

**Table 1.** Sample information for each dataset

Accession	platform	IDD	control
GSE56081	Agilent_033010 Probe Name version	5	5
GSE124272	Agilent-072363 SurePrint G3 Human GE v3 8x60K Microarray 039494	8	8
GSE150408	Agilent-072363 SurePrint G3 Human GE v3 8x60K Microarray 039494	17	17

The probe sequence of the GSE56081 dataset was aligned to the genome by chip reannotation to obtain the transcript ID of the probe mapping, and each transcript cluster was assigned to the Ensembl gene ID. For the transcription clusters with Ensembl gene IDs, clusters with annotation types of ‘lncRNA’, ‘sense_intronic’, ‘sense_overlapping’, ‘antisense’, ‘processed_transcript’, ‘3prime_overlapping_ncRNA’ were considered as lncRNAs [17]. A total of 3501 lncRNAs were finally identified after removing repeated transcripts. In addition, the cluster with the annotation type ‘protein_coding’ were regarded as coding genes, and finally 6991 coding genes were identified. The same pipeline was used to re-annotate the probe sequence of the GSE124272 dataset, and finally 11,733 lncRNAs and 11,520 coding genes were retained.

For mRNA and lncRNA expression profiles, when multiple probes were mapped to the same gene, the median value was taken as the expression value of the gene.

## Differential expression analysis and weight co-expression network

R software package limma [18] was used to screen the differential genes and lncRNAs between normal samples and IDD samples. To obtain biologically different genes, FDR <0.01 and a two-fold difference served as the threshold to detect differentially expressed genes (DEG) and lncRNA (DEL) in GSE56081 dataset. In addition, the expression profiles of lncRNA and genes were combined to construct WGCNA for a better identification of disease-related genes and lncRNA. RNA Expression data profile of genes/lncRNAs was tested to examine the quality of samples and genes/lncRNAs. Then, the WGCNA [19] package in R was used to construct scale-free co-expression network for the genes/lncRNAs. Pearson’s correlation matrices and average linkage method were both performed for all pair-wise s. Then, a weighted adjacency matrix was constructed using a power function (= Pearson’s correlation between gene/lncRNA m and gene/lncRNA n; = adjacency between gene/lncRNA m and gene/lncRNA n). β was a soft-thresholding parameter to address strong correlations between gene/lncRNAs and penalize weak correlations. After choosing the power of β, the adjacency was transformed into a topological overlap matrix (TOM), which could measure the network connectivity of a gene/lncRNA that was defined as the sum of its adjacency with all other gene/lncRNAs for network gene/lncRNA ration. In this way, corresponding dissimilarity (1-TOM) was calculated. To classify gene/lncRNAs with similar expression profiles into gene/lncRNA modules, average linkage hierarchical clustering was conducted according to the TOM-based dissimilarity measure with a minimum size (gene/lncRNA group) of 30 for the gene/lncRNAs dendrogram. The dissimilarity of module eigen gene/lncRNAs was calculated for further analysis of the module, and a cut line was chosen for module dendrogram to merge some modules.

## Identification of disease-related co-expression modules

The module related to the occurrence of IDD was defined as the Co-DGL Module. The genes and lncRNA in the Co-DGL Module were differentially co-expressed genes/lncRNA. Two methods were employed to identify the modules related to the occurrence of IDD. The significance of Gene/lncRNA (CS) was defined as the log10 conversion of the P value (CS = lgP). In addition, module significance (MS) was the average CS of all Gene/lncRNA in the module. Generally, a module with an absolute MS ranked the first or the second among all selected modules is regarded as a module related to clinical traits. Module eigengenes (MEs) are considered to be the main component in the principal component analysis of each Co-DGL Module, and the expression patterns of all Gene/lncRNA could be summarized as a single characteristic RNA expression profile within a given module. In addition, we also calculated the correlation between ME and clinical features to determine relevant modules. The module with the largest absolute MS among all selected modules was generally related to clinical characteristics.

## Functional enrichment analyses

Gene Ontology (GO) and Kyoto Encyclopedia of Genes and Genomes (KEGG) pathway enrichment analysis was performed using the R software package clusterProfiler [20] for genes associated with modules, which were significantly related to the disease, to identify over-represented GO terms in three categories (biological processes, molecular function and cellular component) and KEGG pathway. For both analyses, a P-value of <0.05 denoted statistical significance.

## Regulatory interaction between miRNA-mRNA and miRNA-lncRNA duplex

The miRNA-mRNA regulatory relationships were collected from miRanda [21], miRTarBase [22], TargetScan [23] and starBase [24] databases, and 416,312 non-redundant miRNA–mRNAs interaction were obtained. The miRNA–lncRNA interaction was retrieved from the starBase [25] and miRcode [26] databases, and 295,601 non-redundant miRNA–lncRNA relationships were retained.

## Disease disorder lncRNA-mRNA pairs (LmiRM-Degenerated)

Based on the ceRNA hypothesis [27,28], a candidate LmiRM-Degenerated is defined if it meets all the following conditions: (1) The presence of miRNA shared by mRNA and lncRNA is significantly enriched (determined by hypergeometric test, p < 0.01); (2) mRNA-lncRNA in the same disease-related co-expression module.

## Disease genes and LmiRM-Degenerated network construction

We screened the gene set related to IDD from the DisGeNET v6.0 [29] database, which contained 343 genes in total. Genes and the genes in LmiRM-Degenerated were mapped to string v11.0 [30] dataset to obtain the protein interaction network. The shortest path from each LmiRM-Degenerated gene to the IDD-related gene was further counted, and the shortest path between the IDD-related genes was also compared. The shortest median path between IDD genes was the threshold to determine the relationship between LmiRM-degenerated genes and IDD as a gene specific. We further screened the lncRNAs interacting with IDD-specific genes, and counted interaction frequency between lncRNAs and IDD-specific gene. LncRNAs interacting with more than 50% of IDD-specific genes were determined as IDD-specific lncRNAs.

## Disease-specific gene and construction of drug target network

To determine the potential drug effects of these IDD-specific genes and lncRNAs, we obtained the relationship between drugs and drug target genes from the drugbank v5.1.7 [31] database, and collected 16,196 drug-gene interaction data. Drug target genes and IDD-specific genes were mapped to the string v11.0 database and obtained 40,919 pieces of gene interaction information, and finally a drug-gene-IDD-specific gene network was constructed. The shortest path from each drug to IDD-specific gene in the network was calculated, and the drug with the average shortest path to IDD-specific gene was determined as the candidate treatment drug.

## Construction of IDD diagnostic prediction model and evaluation of model prediction ability

IDD-specific genes and ID-specific lncRNAs were used to construct a diagnostic prediction model based on support vector machine (SVM) [32] classification to predict IDD. In machine learning algorithms, SVM is a supervised learning model that analyzes data and recognizes patterns. SVM, which create a hyperplane in high or infinite dimensional space, can be used for classification, regression. Given a set of training samples, and each tag belongs to two categories, a SVM training algorithm establishes a model and assigns new instances to one category or another so that non-probabilistic binary linear classification was achieved. The model was constructed in the training data set, and the model classification performance was verified by the ten-fold cross-validation method. The established model was then used to predict the samples in the validated data set. The predictive ability of the model was evaluated by area under ROC curve (AUC), moreover, the predictive sensitivity and specificity of the model to IDD were analyzed.

## Results

The purpose of this study was to use the RNA expression profiles from IDD patients to study the potential functions of lncRNA and mRNA expression in IDD. We systematically analyzed the lncRNA and miRNA expression profiles between IDD and healthy patients. Finally, a genetic diagnosis model and lncRNA diagnosis model, which were established based on these genes and lncRNAs, showed 100% diagnostic performance in both the training data set and the validation data set.

## Identification of DEGs/DELncRNAs

**Figure 2.**
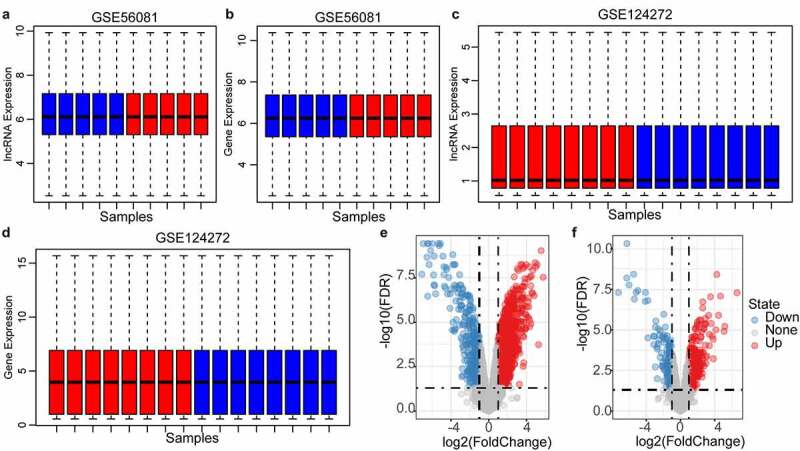
Identification of differentially expressed genes. A: the expression distribution of protein coding genes in each sample in the GSE56081 dataset; B: the expression distribution of lncRNAs in each sample in the GSE56081 dataset; C: the protein coding genes in each sample in the GSE124272 dataset D: the expression distribution of lncRNAs in each sample in the GSE124272 data set; where red represents disease samples and blue represents healthy samples; E: GSE56081 data set protein coding gene difference volcano map, F: GSE56081 data set lncRNA Difference volcano map

Updated gene and lncRNA signatures could be obtained by reannotating the microarray using the latest genomic information. After data standardization and chip re-annotation, the expression profiles of 6991 genes and 3501 lncRNAs were finally screened from the GSE56081 dataset (Figure 2(a, b), FDR<0.01), and the expression levels of lncRNAs and protein-coding genes in each sample were similar. The expression profiles of 11,733 lncRNAs and 11,520 coding genes were also screened from the GSE124272 dataset (Figure 2(c, d), FDR<0.01), and the median expression level of lncRNA was found to be significantly lower than that of coding genes. Therefore, GSE56081 was used as the training set, and GSE124272 was used as the verification data set. In the training set, we identified a total of 1455 differentially expressed genes and 423 differentially expressed lncRNAs (Figure 2(e, f), P < 0.05).

## Construction weighted co-expression network and identification of disease-related module

**Figure 3.**
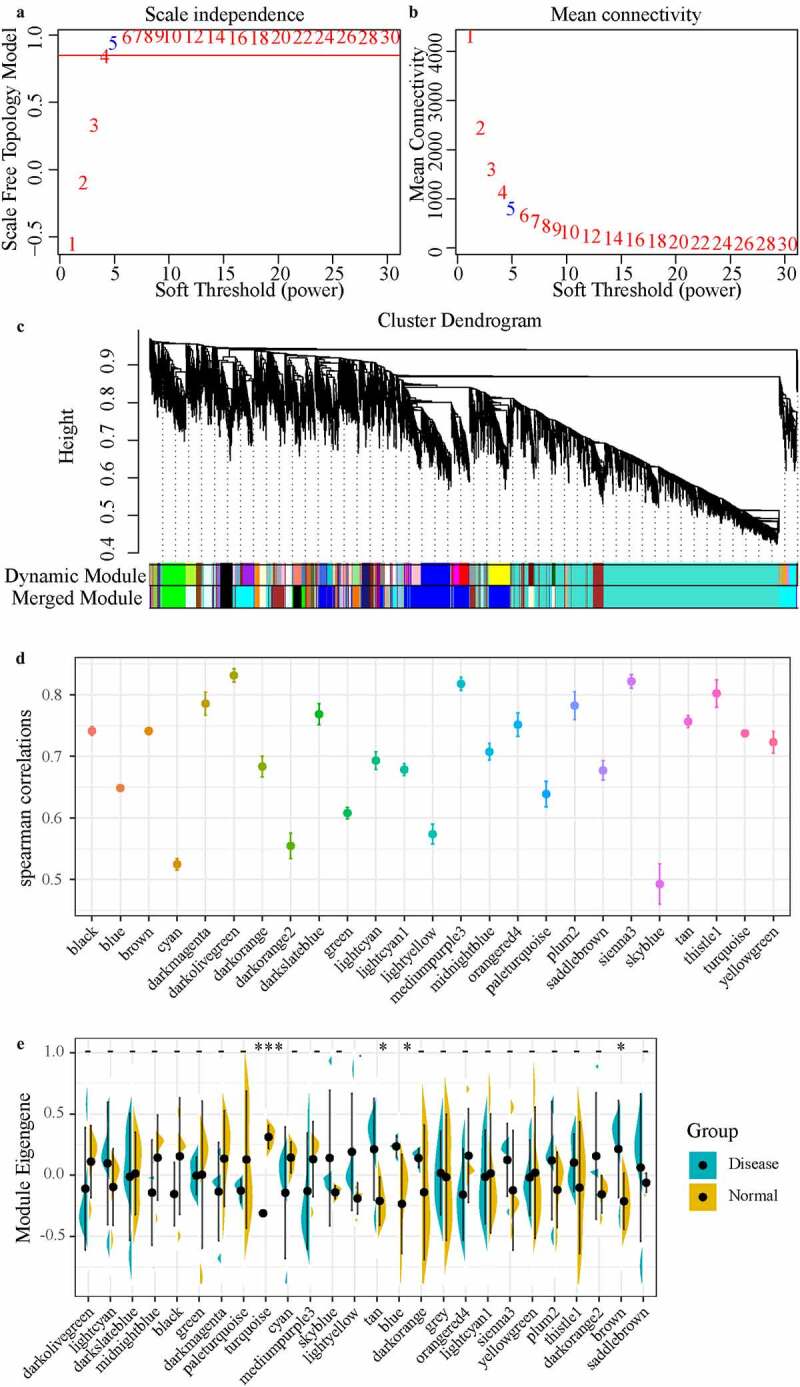
Weighted co-expression network construction and disease-related module identification, (AB) Determination of soft-thresholding power in the weighted gene co-expression network analysis (WGCNA). (a) Analysis of the scale-free fit index for various soft- thresholding powers (β). (b) Analysis of the mean connectivity for various soft-thresholding powers. (c) Dendrogram of all differentially expressed genes/lncRNAs clustered based on a dissimilarity measure (1-TOM). (d) Distribution of average gene significance and errors in the modules associated with the Degenerated. (e) The distribution of the feature vector of each module in the Degenerated and healthy control samples

In a biological system, specific functional regulation is often co-participated by one or more genes, and these genes have certain similarities in expression. Therefore, gene sets involved in certain functions could be identified through co-expression analysis. In this study, the power of β = 5 (no scale = 0.94) was the soft threshold to ensure a scale-free network (Figure 3(a, b)). A total of 26 modules were identified (Figure 3(c)). To determine correlation of the disease and module, the Spearman correlation coefficient was calculated between gene/lncRNA and disease occurrence in each module (Figure 3(d)), and module with the median value of correlation coefficient greater than 0.7 were selected. In disease and health groups, the differences in the distribution of feature vectors across modules showed that the distribution of feature vectors in the disease group was significantly higher in the tan, blue, and brown modules than in the health group (Figure 3(e)). Based on these two methods, the tan, brown, and turquoise modules closely related to the occurrence of the disease were identified as the key modules of IDD. Where the tan module contains 80 lncRNAs and 150 genes, the brown module contains 291 lncRNAs and 552 genes, and turquoise contains 1263 lncRNAs and 2939 genes, with no intersection of genes and lncRNAs in the three modules, where the turquoise module was negatively correlated with brown and tan modules, and a weak positive correlation was shown between tan and brown modules (Figure S1A).

## Functional implications of degeneration-related module

**Figure 4.**
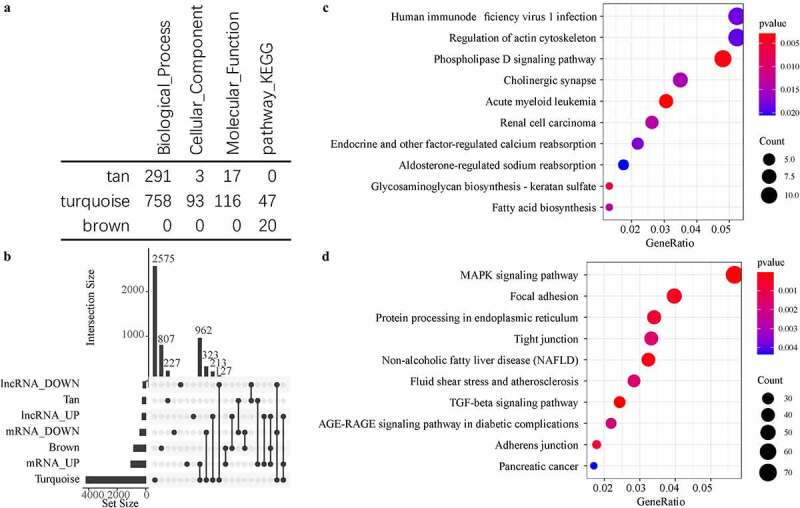
Functional enrichment analysis of disease-related modules. A: GO Term and KEGG Pathway statistics enriched by the three modules; B: Venn diagram of the intersection between enriched genes and lncRNAs of the three modules and differential genes and lncRNAs; C: the most significant enrichment of the brown module Top 10 KEGG Pathway. D: The most significant top 10 KEGG Pathway enriched by the turquoise module. Different colors indicate the significance of enrichment, and the size of the dot indicates the number of enriched genes

Functional enrichment analysis of gene sets effectively identifies dysfunctional pathways. To better understand the functional implications of the three disease-related modules, GO and KEGG functional enrichment analysis was performed on the genes in the three modules. We observed that these three modules were enriched in a large number of GO terms and KEGG pathways (Figure 4(a)), and that the generation tan module was mainly enriched in a large number of biological processes. The brown module was found to be more enriched in KEGG Pathway, and turquoise module was enriched in a variety of biological processes, molecules function, and signaling pathways. We also counted the intersection of genes and lncRNAs in these three modules with differentially expressed genes and differential expressed lncRNAs (Figure 4(b)). It was also found that the turquoise module contained a large number of differentially expressed genes and differentially expressed lncRNAs, which were hardly identified in the tan module, moreover, brown contained a small amount of differential expressed genes and differential expressed lncRNAs. Further analysis of KEGG pathways enriched by Turquoise and Brown modules showed that the brown module was primarily enriched with phosphoolipase D signaling Pathway, Cholinergic synapse, other factor-regulated calcium reabsorption pathways, and some other pathways (Figure 4(c)), and that the turquoise module was mainly enriched in MAPK signaling pathway, TGF-beta signaling pathway, AGE-RAGE signaling pathway in diabetic complications and other signaling pathways (Figure 4(d)). Interestingly, TGF-beta signaling pathway the most significant signaling pathway has activation effect against Wnt signaling pathway, and its abnormality will lose the antagonistic effect against Wnt signaling pathway, resulting in difficulties in IDD repair and accelerating degeneration.

## Identification of LmiRM-Degenerated

**Figure 5.**
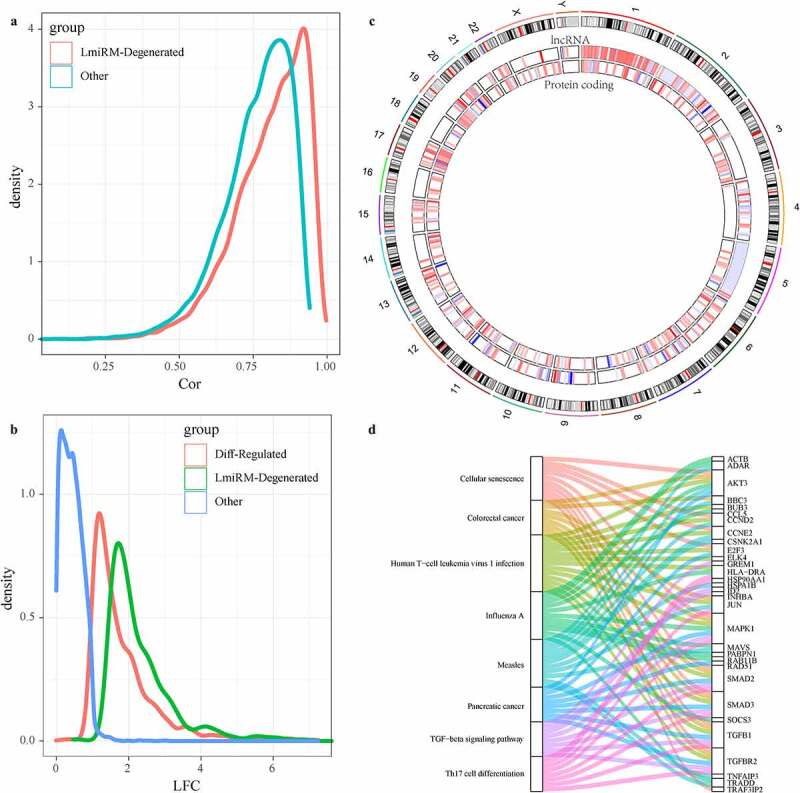
Identification of LmiRM-Degenerated and its role in intervertebral disc degeneration. A: Comparison of the correlation between lncRNA-mRNA in LmiRM-Degenerated and the correlation distribution of non-LmiRM-Degenerated lncRNA-mRNA; B: LmiRM-Degenerated Comparison of the fold of expression difference between the RNA and non-LmiRM-Degenerated differential RNA and non-differential RNA; C: the distribution of lncRNA and mRNA in LmiRM-Degenerated on the genome, the color of the inner circle heat map gradually changes from blue to red Represents the expression difference multiples from low to high; D: LmiRM-Degenerated enriched KEGG Pathway and gene relationship circle diagram, the right side is the pathway, different colors indicate different pathways, the left is the gene, and different colors indicate expression difference multiple

The ceRNA(competing endogenous RNAs) hypothesis reveals a new mechanism of interaction between RNAs, which represents a new regulation mode of gene expression. Here, we developed a new calculation method to identify LmiRM-Degenerated in IDD. Gene/lncRNA matching expression profiles from disease-related co-expression modules were integrated into the Gene Expression Omnibus (GEO) data set based on the regulatory interactions among mRNAs, lncRNAs and miRNAs. Here, 11,857 LmiRM-Degenerated containing 352 mRNAs and 245 lncRNAs were obtained. The role of these LmiRM-Degenerated in IDD was examined from multiple perspectives. Firstly, the correlation distribution of mRNA-lncRNA in LmiRM-Degenerated was significantly higher than that of random differentially expressed RNAs of the same module (Figure 5(a)), which suggested that these mRNA-lncRNA interacted more closely and actively. After analyzing the multiple distribution expression of mRNAs and lncRNAs in Lmirm-degenerated, we observed that these lncRNAs and mRNAs had a higher expression differential multiples when compared with the differentially expressed mRNAs and lncRNAs (Figure 5(b)), indicating that mRNAs and lncRNAs in LmiRM-degenerated showed more obvious changes in disease samples. Furthermore, we analyzed the distribution of these lncRNAs and mRNAs in the genome (Figure 5(c)), and the data showed that lncRNAs tended to concentrate on chr1, chr2, chr3, while mRNA tended to concentrate on chr17, chr19, and chr20. In addition, from KEGG Pathway enrichment analysis, it could be found that these LmiRM-Degenerated were related with colorectal cancer, pancreatic cancer, TGF-beta signaling Pathway, Th17 cell differentiation, cellular senescence, influenza A, measles, human T-cell leukemia virus 1 infection (Figure 5(d)). As ceRNA analysis is still an evolving field. The latest study of ncRNAs associated with IDD through accurate transcriptional spectrogram analysis may contribute to the discovery of clinical significance of other LmiRM-Degenerated.

## LmiRM-Degenerated analysis revealed biomarkers for the diagnosis and treatment of IDD

**Figure 6.**
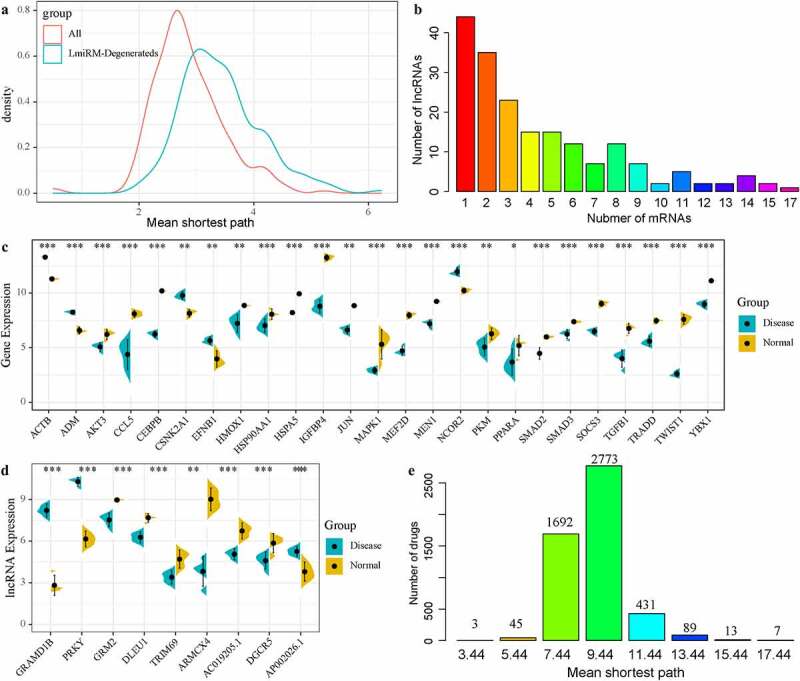
LmiRM-Degenerated analysis revealed biomarkers for the diagnosis and treatment of intervertebral disc degeneration. A: Disease genes in the interaction network between disease genes and LmiRM-Degenerateds genes-the shortest path distribution of disease genes and disease genes -LmiRM- The shortest path distribution between Degenerateds genes. B: Frequency statistics of lncRNAs that interact with disease-specific genes. The x-axis is the number of disease-specific genes corresponding to the lncRNA, and the y-axis is the frequency of lncRNA. C: The differential expression distribution of 25 disease-specific genes. D: The differential expression distribution of 9 disease-specific lncRNAs. E: The average shortest path distribution from the drug to the IDD-specific gene

To determine the potential diagnostic and prognostic markers of IDD, we used linear discriminant analysis to classify and predict each LmiRM-Degenerated. We observed that 11,092 (93.5%) lmiRM-degenerateds could predict patients with 100% accuracy, suggesting that these LmiRM-Degenerateds were potential diagnostic markers of disc degeneration. Furthermore, gene set related to IDD were screened from the DisGeNET v6.0 [29] database. After mapping these genes set and the coding genes in LmiRM-Degenerateds to the string database, a total of 2258 interactions were obtained. A network of LmiRM-Degenerateds and disease gene regulation interactions was established. The shortest path between each LmiRM-Degenerated and disease gene in the network and between two disease genes in the network were separately calculated. By comparing the shortest path distribution of the two, it was observed that there were significant differences in the average shortest path between the two, and a shortest path between disease genes was identified (Figure 6(a)), which indicated that the regulation of disease genes was closer, and the shortest path between disease genes was more related to IDD. Based on this, we determined the mean shortest path between Lmirm LmiRM-Degenerated and disease genes shorter than the median of the shortest path between disease genes and disease genes as the threshold to identify new potential disease-related genes. Under such a condition, we obtained a total of 25 genes, of which 6 have been reported to be related to IDD. Further, LmiRM-degenerateds of these 25 genes were screened, and it was observed that most lncRNAs had a low frequency, while a few lncRNAs had a high frequency, moreover, lncRNAs with a high frequency were more closely related to the interactions of these 25 genes (Figure 6(b)). Finally, we selected a total of 9 lncRNAs with occurrence frequency was greater than 12. Among the 25 genes, 5 genes were down-regulated and 20 genes were up-regulated (Figure 6(c), p < 0.05); 6 lncRNAs were down-regulated, 3 lncRNAs were up-regulated (Figure 6(d), p < 0.05). In addition, we analyzed the shortest path distribution of 25 diseases specific genes from drugs in the network. The average shortest path of most drugs was 9.44, and the average shortest path of three drugs (Calcium citrate, Calcium phosphate, Calcium phosphate dihydrate) was only 3.44 (Figure 6(e)), suggesting that the three drugs might have therapeutic effects on IDD.

## Advantages of diagnostic models

**Figure 7.**
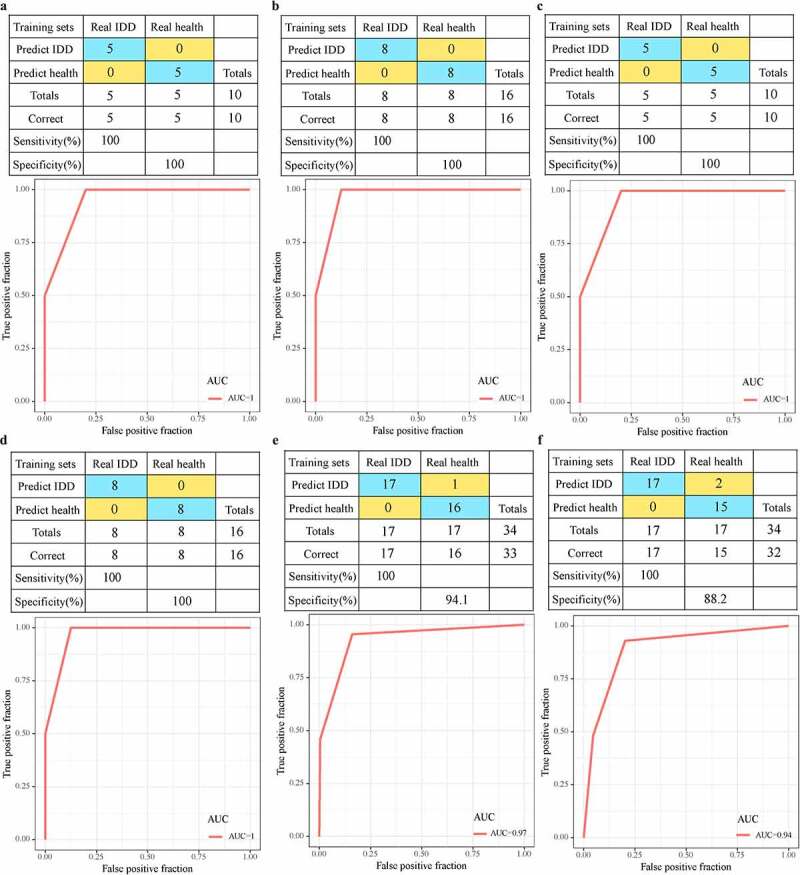
Advantages of diagnostic models. A: The classification results and ROC curve of the samples of the genetic diagnosis model in the training dataset; B: The classification results and ROC curve of the samples of the genetic diagnosis model in the validation dataset; C: The classification results and ROC curve of the lncRNA diagnosis model in the training dataset; D: The classification results and ROC curve classified by the lncRNA diagnosis model in the validation dataset. E: The classification results and ROC curve of the samples of the genetic diagnosis diagnosis model in the GSE150408 dataset. F: The classification results and ROC curve of the samples of the lncRNA diagnosis model in the GSE150408 dataset

Considering that genes detected by different chip platforms are different from lncRNAs, we selected 19 genes and 7 lncRNAs detected both in the training set and the validation set as features to construct a diagnostic model. In the training data set, 19 genes were used as features to construct the SVM classification model, and the model test was carried out using the ten-fold cross-validation method, with 100% classification accuracy. The sensitivity and specificity of the model to IDD were all 100%, and the area under ROC curve (AUC) was 1.0 (Figure 7(a)). The GSE124272 data set was further used for verification. Among the 16 samples, 16 were all correctly classified, with a classification accuracy of 100%, the sensitivity and specificity of the model to IDD of 100%, and the area under the ROC curve of 1.0 (Figure 7(b)). In addition, the classification accuracy of 7 lncRNAs in the training set was 100%, the sensitivity and specificity of the model to IDD were 100%, and the area under ROC curve (AUC) was 1.0 (Figure 7(c)). Among the 16 samples in the validation set, 16 samples were correctly classified, with a classification accuracy of 100%, model sensitivity to IDD of 100%, specificity of 100%, and area under ROC curve of 1.0 (Figure 7(d)). Nineteen genes were used as features in the GSE150408 dataset, their corresponding expression profiles were obtained, and the classification accuracy was observed to be 100%, and 33 out of 34 samples were correctly classified. The sensitivity and specificity of the model for IDD were both 100%, and the area under ROC curve (AUC) was 1.0 (Figure 7(e)). In addition, 7 lncRNAs were acted as features, and the classification accuracy in the training set was 100%, and 32 out of 34 samples were correctly classified. The sensitivity and specificity of the model for IDD were both 100%, and the area under the ROC curve (AUC) was 1.0 (figure 7(f)). These results indicated that the diagnostic prediction model constructed in this study can effectively distinguish IDD patients from healthy controls. These genes and lncRNA can be used as reliable biomarkers for IDD-specific diagnosis.

## Discussion

Cervical spondylosis, lumbar disc herniation and other spinal degenerative diseases caused by IDD are chronic diseases that affect the quality of life of middle-aged and elderly people [24,33]. The intervertebral disc tissue is composed of outer fibrous annulus, central nucleus pulposus, and upper and lower cartilage endplates. Under normal circumstances, nucleus pulposus bears axial load and converts it into peripheral tension load. The annulus fibrosus absorbs these stresses and maintains the stability of intervertebral disc [34]. The difference in the structure and function of annulus fibrosus and nucleus pulposus determines the pathological changes of the two during IDD, but the genes and lncRNA backgrounds underlying such difference are still unclear. In this study, the differences in gene expression and lncRNA expression between IDD and healthy samples were systematically analyzed, and the lncRNAs and genes were reconfirmed through the weighted co-expression method. The results showed that these genes were mainly enriched in MAPK signaling pathway, TGF – among various signaling pathways such as beta signaling pathway and AGE-RAGE signaling pathway in diabetic complications. TGF-beta signaling pathway as the most significant pathway has the effect of antagonizing the activation of Wnt signaling pathway, and its abnormality will lose antagonism to Wnt signaling pathway, resulting in difficulties in repairing IDD and accelerating degeneration.

In addition, this study systematically analyzed IDD-related gene and lncRNA expression data through using a new calculation method that integrates sample-matched mRNA and lncRNA expression profiles, and discovered dysregulated ceRNA triad. Dynamic expression analysis was performed through microarray re-annotation, and lncRNA and mRNA expression profile data were obtained. There is evidence that about 10% to 30% of the microarray probes designed for protein-coding genes are actually mapped to non-coding RNAs [35], which can be collected by re-annotation. The expression information of lncRNA is a commonly used method in transcription studies [36,37]. According to the previously described pipeline [38], we 2directly extracted the expression data of lncRNA from the existing expression profile to reduce errors. After differential expression and co-expression analysis, the dysregulated lncRNAs and mRNAs in IDD were determined, and then the dysregulated LmiRM-Degenerateds were obtained. We also observed that the correlation distribution of mRNA-lncRNA in LmiRM-Degenerated was significantly higher than that of the same random modules. These results indicated that mRNA-lncRNA in LmiRM-Degenerated had stronger expression correlation and expression changes. In addition, the KEGG Pathway enrichment analysis of LmiRM-Degenerated showed that these LmiRM-Degenerateds were related to TGF-Beta signaling pathway, Th17 cell differentiation, and Human T-cell leukemia virus 1 infection, indicating that the occurrence of IDD was a complicated process associated with TGF-beta and immune processes.

We constructed an interaction network between disease genes and LmiRM-Degenerated with the reported IDD-related gene sets, analyzed the shortest path distribution of each LmiRM-Degenerated gene and known IDD-related genes. In this way, 25 new IDD-specific expression gene sets were determined, and 6 of these 25 genes, such as ACTB, HMOX1, JUN, MAPK1, SMAD3, TGFB1, have been reported to be associated with IDD, and there were 9 IDD-specific lncRNAs (GRAMD1B, PRKY, GRM2, DLEU1, TRIM69, ARMCX4, AC019205.1, DGCR5, AP002026.1). We downloaded the drug and gene interaction data from the Drugbank database to construct a drug-target gene-disease-specific gene interaction network. The method with the shortest path determined that the three drugs Calcium citrate, Calcium phosphate, and Calcium phosphate dihydrate may have curative effects on IDD. SVM was employed to construct and verify the expression profiles of these disease-specific lncRNA and mRNA classifiers, and the AUC reached 1 in both the training set and the validation set, showing that these genes and lncRNAs had a high classification effect on IDD. We also examined the expressions of those lncRNAs on IDD tissues using RT-qPCR, and the results showed that GRAMD1B expression was upregulated, while the levels of DLEU1, ARMCX4, AC019205.1, DGCR5 were downregulated in IDD tissues (Figure S2).

Although we have systematically analyzed the abnormal expression and function of mRNAs and lncRNAs in IDD through bioinformatics techniques, attention should also be paid to some limitations of this research. Firstly, the sample lacked some clinical follow-up information, therefore, we did not consider factors such as the presence of other health status of the patient when distinguishing these biomarkers. Secondly, the results obtained through only bioinformatics analysis were not fully convincing, and experimental verification was needed to confirm the present results. Therefore, further genetic and experimental research with larger sample size and experimental verification is needed.

## Conclusion

In conclusion, in this study, we systematically analyzed the expression changes of lncRNAs and genes in IDD, and conducted a large-scale genome-wide study on RNA expression profiles. Based on the characteristics of 25 genes and 9 lncRNAs in IDD, we found that these dysregulated lncRNAs and genes were involved in a variety of important biological pathways of IDD. At the same time, we also observed that three drugs, Calcium citrate, Calcium Phosphate, and Calcium phosphate dihydrate, may be effective in IDD treatment, providing useful targets and references for future studies.

## Supplementary Material

Supplemental Material

## Highlights

1. We identified 1455 differentially expressed genes and 423 differentially expressed lncRNAs.

2. Twenty-six co-expression modules were obtained, among them, the tan, brown, and turquoise modules are most related to IDD.

3. By constructing a disease–gene interaction network, 25 disease-specific genes and 9 disease-specific lncRNAs were identified.
