# Supplementary figures and images for: Systematic analysis of Long non-coding RNAs reveals diagnostic biomarkers and potential therapeutic drugs for intervertebral disc degeneration
Source: Bioengineered. 2021 Aug 17;12(1):5069–84. doi: 10.1080/21655979.2021.1950258 (PMC8806434; doi:10.1080/21655979.2021.1950258)

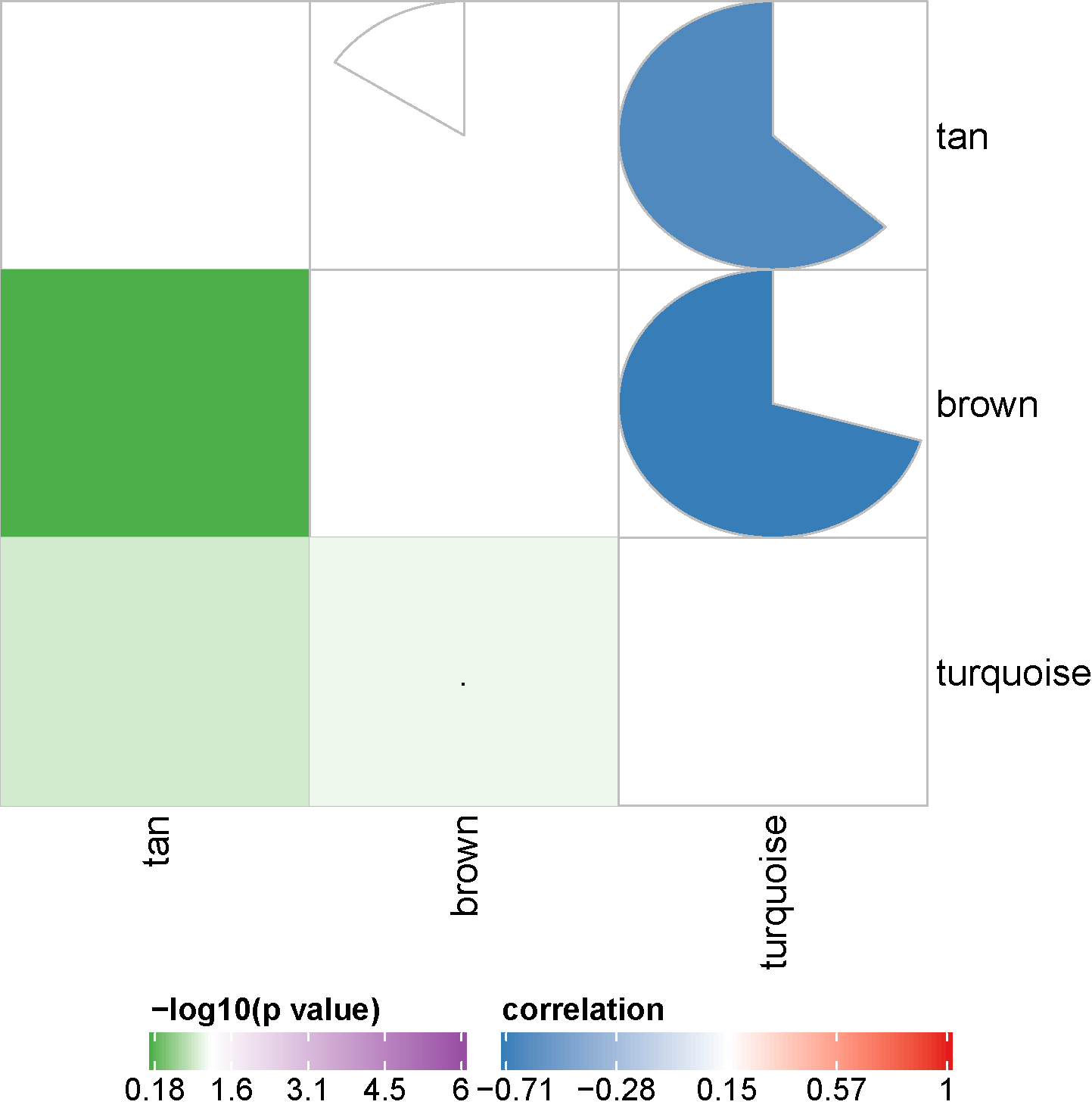

Supplement: Supplemental Material [file KBIE_A_1950258_SM7749.zip › supplementary/Figure S1.tif]

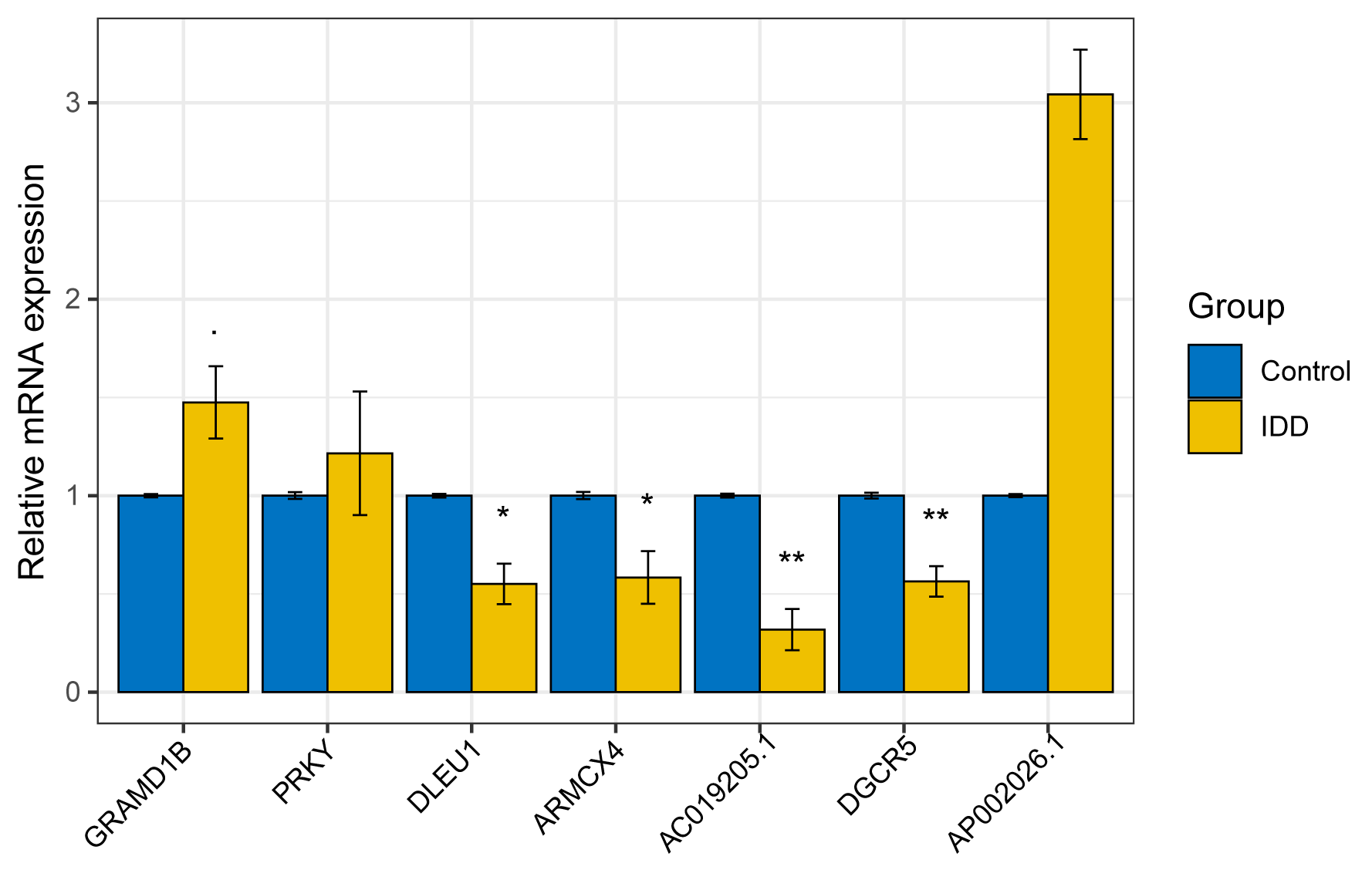

Supplement: Supplemental Material [file KBIE_A_1950258_SM7749.zip › supplementary/Figure S2.tif]
